# Surgical Management of Perianal Giant Condyloma Acuminatum of Buschke and Löwenstein: Case Presentation

**DOI:** 10.3390/life13091916

**Published:** 2023-09-15

**Authors:** Raul Mihailov, Alin Laurențiu Tatu, Elena Niculet, Iulia Olaru, Corina Manole, Florin Olaru, Oana Mariana Mihailov, Mădălin Guliciuc, Adrian Beznea, Camelia Bușilă, Iuliana Laura Candussi, Lavinia Alexandra Moroianu, Floris Cristian Stănculea

**Affiliations:** 1 Faculty of Medicine and Pharmacy, Dunărea de Jos University, 800008 Galati, Romania; raul.mihailov@ugal.ro (R.M.); alin.tatu@ugal.ro (A.L.T.); elena.niculet@ugal.ro (E.N.); adrianbeznea@hotmail.com (A.B.); camelia.busila@ugal.ro (C.B.); iuliana.candussi@ugal.ro (I.L.C.); lavinia.moroianu@ugal.ro (L.A.M.); 2 Clinical Emergency County Hospital Sf. Ap. Andrei, 800578 Galați, Romania; iulia_dabija@yahoo.com (I.O.); florin.olaru@yahoo.com (F.O.); 3 Dermatology Department, Saint Parascheva Infectious Diseases Clinical Hospital, 800179 Galați, Romania; 4 Multidisciplinary Integrated Center of Dermatological Interface Research Center (MICDIR), “Dunărea de Jos” University of Galați, 800201 Galați, Romania; 5 ‘Sf. Ioan’ Clinical Hospital for Children, 800487 Galati, Romania; 6 Faculty of Medicine and Pharmacy, “Carol Davila” University, 020021 Bucharest, Romania; floris-cristian.stanculea@drd.umfcd.ro

**Keywords:** Buschke–Löwenstein, condyloma acuminatum, reconstructive surgery, skin surgery

## Abstract

Introduction: The Buschke–Löwenstein tumor (BLT) is an uncommon sexually transmitted ailment attributed to the human papillomavirus (HPV)—usually the 6 or 11 type (90%)—with male predominance and an overall infection rate of 0.1%. BLT or giant condyloma acuminatum is recognized as a tumor with localized aggressiveness, displaying distinctive features: the potential for destructive growth, benign histology, a rate of 56% malignant transformation, and a high rate of recurrence after surgical excision. There are several treatment choices which have been tried, including laser, cryotherapy, radiotherapy, electrocoagulation, immunotherapy, imiquimode, sincatechins, intralesional injection of 5-fluoruracil (5-FU), isolated perfusion, and local or systemic chemotherapy. In the case of an extensive tumor, preoperative chemotherapy or radiotherapy is used for tumor shrinkage, making the debulking procedure safer. HPV vaccines significantly decrease the incidence of genital warts, also decreasing the risk of BLT; HPV-6 and HPV-11 are included in these vaccines. Materials and methods: We present a 53-year-old heterosexual man, hospitalized in our department in June 2021 with a typical cauliflower-like tumor mass involving the perianal region, which progressively increased in size for almost 7 years. The perianal mass was completely removed, ensuring negative surgical margins. The large perianal skin defect which occurred was reconstructed with fascio-cutaneous V-Y advancement flap. There was no need for protective stoma. The literature review extended from January 1980 and December 2022, utilizing Pubmed and Google Scholar as search platforms. Results: Due to the disease’s proximity to the anal verge and the limited number of reported cases, arriving at a definitive and satisfactory treatment strategy becomes challenging. The optimal approach entails thorough surgical removal of the lesion, ensuring well-defined surgical margins and performing a wide excision to minimize the likelihood of recurrence. In order to repair the large wound defects, various rotation or advancement flaps can be used, resulting in reduced recovery time and a diminished likelihood of anal stricture or other complications. Our objective is to emphasize the significance of surgical excision in addressing BLT through the presentation of a case involving a substantial perianal condyloma acuminatum, managed successfully with complete surgical removal and the utilization of a V-Y advancement flap technique. In the present case, after 5 months post operation, the patient came back with a buttock abscess, which was incised and drained. After another 5 months, the patient returned for difficult defecation, with an anal stenosis being diagnosed. An anal dilatation and sphincterotomy were carried out, with good postoperative results. Conclusions: The surgical management of Buschke–Löwenstein tumors needs a multidisciplinary team with specialized expertise. The reconstruction techniques involved can be challenging and may introduce additional complications. We consider aggressive surgery, which incorporates reconstructive procedures, as the standard treatment for Buschke–Löwenstein tumors. This approach aims to achieve optimal surgical outcomes and prevent any recurrence.

## 1. Introduction

The precise incidence and prevalence of BLT are difficult to establish due to a lack of standardized diagnostic criteria; by individually documenting multiple cases, an estimated overall prevalence of 0.1% within the general population is determined, most cases developing between the fourth and sixth decade of life [1,2,3,4,5]. Additionally, a few cases have been described in the pediatric population [6]. The mean age reported by L. J. Trombetta and R. J. Place is 43.9 years old, with a male-to-female ratio of 2.7 to 1, with a few cases described in pregnant women [7,8]. HPV is the main agent in BLT pathogenesis, with subtypes 6 and 11 contributing to over 90% of cases in both immunocompetent individuals and those with immunosuppressive conditions [9,10]. It has been reported that individuals who engage with numerous sexual partners, have chronic genital infections, maintain suboptimal hygiene practices, or suffer from immunodeficiencies are at greater risk of this condition [9,10]. The role of HPV vaccination has been proven to be protective, but due to the rarity of the disease, research regarding the treatment and prevention of BLT remains limited [11].

## 2. Materials and Methods

The literature search encompassed the time span from January 1980 to December 2022 and was conducted on Pubmed and Google Scholar. English was selected as the preferred language for publication. The keywords used were Buschke–Löwenstein and surgery. We excluded articles in non-adult populations. The selection criterion was studies that include clinical data and the therapeutic management (especially surgical) of BLT.

## 3. Results

### 3.1. Clinical Presentation

Trombetta and Place outlined the clinical manifestations of BLT, with the primary indicator being a perineal mass [7]. Dysuria, abdominal distention, fatigue, constipation, difficulty defecating, difficulty urinating, and hemorrhoids were documented by Q. D. Chu and M. P. Vezeridis [1]. Differential diagnosis includes squamous cell carcinoma, squamous cell epitheliomas, inguinal granuloma, anogenital amebiasis, Nicolas Favre disease, secondary syphilis, vegetative tuberculosis, and transmitted diseases such as hepatitis B, hepatitis C, or HIV [5]. 

The tendency of BLT to infiltrate the surrounding tissues and its high risk for malignant transformation make imaging techniques such as computed tomography and magnetic resonance imaging necessary means for assessing both the local and systemic extent [11]. 

There are two different perspectives for confirming BLT malignancy: the first consists of neoplastic histological confirmation, and the other is malignant behavior, which indicates the encroachment into neighboring deeper tissues, even in the absence of the histological verification of malignancy. Chu et al. estimate a rate of malignant transformation of 56% [1]. In their series comprising 51 patients, Trombetta and Place documented the neoplastic occurrence in cases of giant condyloma acuminata, accounting for 58% of the cases, of which 50% were verrucous carcinoma, squamous cell carcinoma, or basal cell carcinoma [7], and 8% corresponded to carcinoma in situ. Prasad and Abcarian documented the neoplastic change in anal condyloma to 1.82%, with four cases exhibiting both histological and invasive characteristics, while only two cases displayed significant infiltration into nearby soft tissues without histological indications of malignancy [12,13,14]. When squamous cell carcinoma occurs in the context of giant condyloma acuminata, management must be chosen by a multidisciplinary team, and there are some cases when only palliative measures can be offered [15,16,17].

### 3.2. Therapeutic Management

There are a lot of treatment options, which include intralesional injection of 5-fluoracil, cryotherapy, interferon, curettage, CO_2_ laser vaporization, interferon, chemotherapy, radiation therapy, and wide surgical resection, alone or with chemotherapy [1]. Despite these multiple treatment options, none of them are more effective than the others, and the high rate of local and regional recurrence demonstrates their inefficiency. Surgical intervention accompanied by clear margins and reconstruction stands as the gold standard and should be executed whenever feasible [15].

Lee’s study demonstrated the efficacy and value of a combined regimen involving topical imiquimod, podophyllotoxin, and cryotherapy for managing extragenital giant condyloma acuminata cases, especially when surgical options are constrained by functional considerations [18,19].

Surgical treatment represented by resection with free margins is the gold standard for BLT. There are many combinations of treatment associated with surgery in an adjuvant or neoadjuvant way, with different results [20]. A uniform method for choosing the treatment approach is absent; instead, it is advisable to create an individualized therapeutic strategy for each case [21].

There are multiple surgical approaches for BLT resection, which can be seen as alternatives, such as radiofrequency, electrocoagulation, carbon laser, or classical surgery. Complete resection is very important, with reconstruction at the same time or in a delayed manner [22]. Surgical removal with well-defined secure margins, ensuring a distance of at least 1 cm, and encompassing the subcutaneous fatty tissues with meticulous dissection from the external anal sphincter is very important. There are many ways to reconstruct the excised area: fascio-cutaneous V-Y advancement flaps, S-flaps, or island-flaps for lesions extending to the perineum to support V-Y flaps for a tension-free reconstruction [14,23,24]. Some reports have indicated the advantages of preoperative selective angioembolization of the internal iliac arteries feeding branches in cases of giant BLT. This approach aims to reduce vascularity and minimize blood loss during the removal of such tumors [25].

In cases of neoplastic transformation to squamous cell carcinoma in BLT, surgical resection can be coupled with radiotherapy and chemotherapy, either as neoadjuvant or adjuvant treatments. The primary protocols of choice involve the utilization of 5-fluorouracil and mitomycin [6,26].

Topical therapies are generally regarded as ineffective due to their elevated failure rate; however, topical imiquimod, which acts as an aminoquinolone modifier of the immune response, has demonstrated advantages in addressing BLT, bucking the trend of ineffectiveness. Initially, a combination of topical imiquimod and CO_2_ laser ablation for residual lesions was documented, subsequently paving the way for its integration with other therapeutic approaches [10,24,27].

### 3.3. Clinical Evolution

The overall recurrence reported by Chu et al. in their study was 67%; approximately half of the patients who underwent surgical intervention as their primary therapeutic approach experienced a recurrence [1]. The duration of the disease is longer in patients with recurrence in contrast to individuals who did not experience recurrence, and those who had an average interval of 10 months until the recurrence occurred [1]. Relapses can be attributed to various factors, such as reinfections, either for the same or different partners, virus reactivation after an extended incubation period, and the inability to completely eliminate lesions containing the virus with current therapies. In such circumstances, unacknowledged and untreated sites may result in recurrences [1,10,17].

The morbidity observed in BLT patients manifests through issues such as the infiltration or infection of soft tissues, recurrences, abscesses, flap failure, fistulas, urethral obstruction, fecal incontinence, anal stenosis, or urinary tract infection [1].

Overall, in the study of Chu et al., the mortality was 21%, associated with morbidities and recurrences, and neoplastic histological transformation did not mean a poor prognosis, having a decreased mortality rate compared to those without the condition [1].

There are no studies which report quality of life associated with BLT. Additionally, the treatment impact, its recurrences, or morbidity are not reported in existing surveys [28,29]. It is considered that this disease, because of its morbidity and the signs and symptoms associated with surgical management, the need for chemotherapy or radiotherapy, and the high rate of recurrence, has a negative influence on patients’ quality of life [30]. There are a few case reports that emphasize the social stigma caused by BLT and the importance of the psychological and emotional impact on these patients [21,22,31].

### 3.4. Case Report

**Figure 1:**
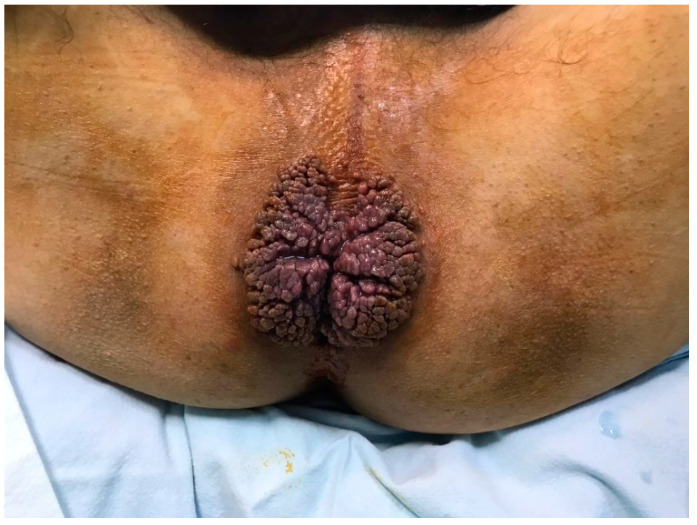
View of the lesion on diagnosis.

**Figure 2:**
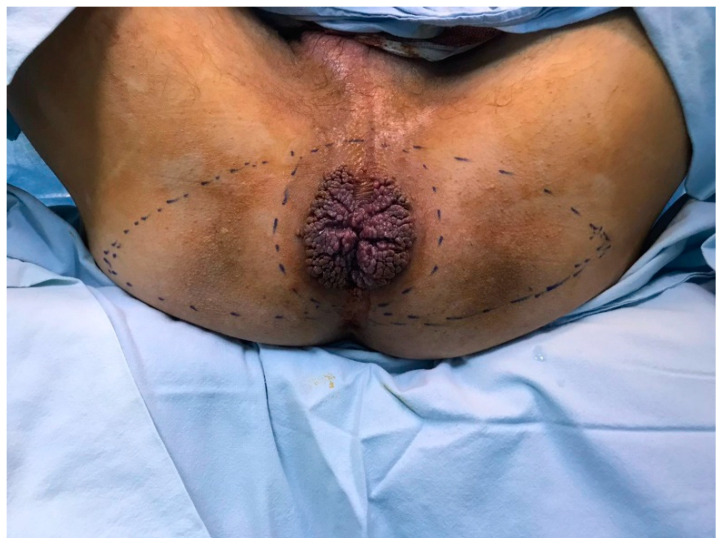
Operative reconstructive plan with V-Y advancement flap.

**Figure 3:**
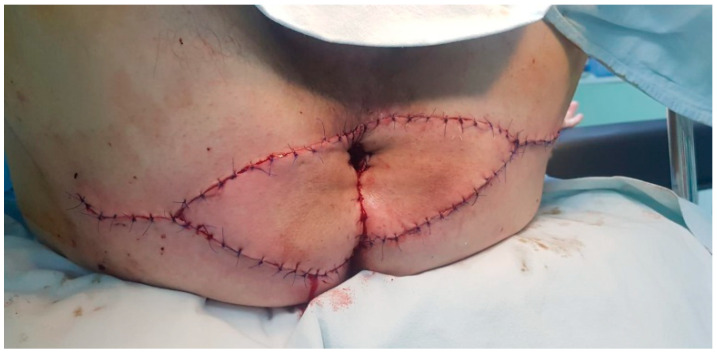
Immediate postoperative condition.

**Figure 4:**
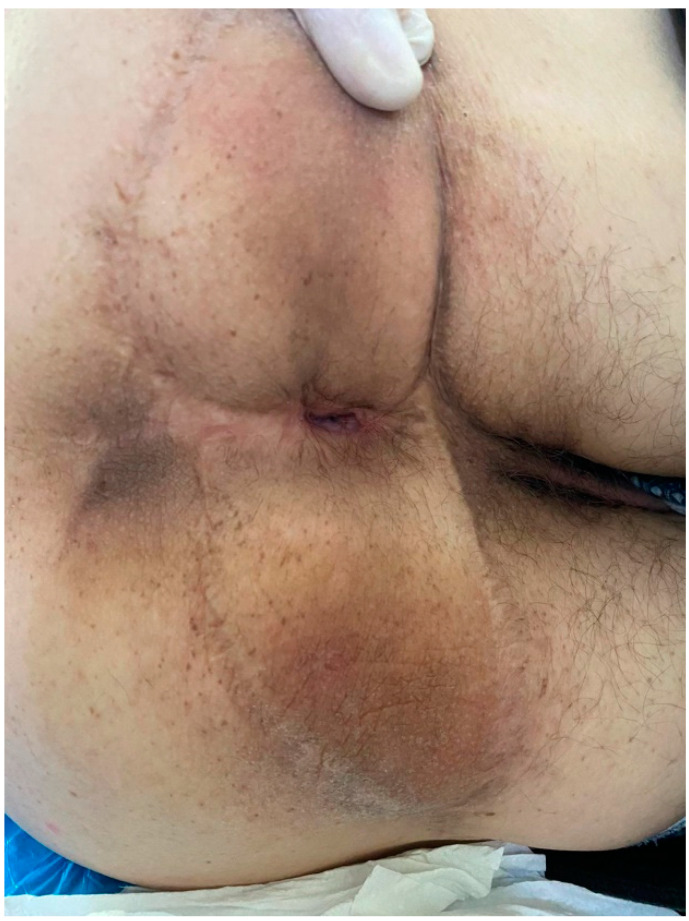
Two years post operation.

**Figure 5:**
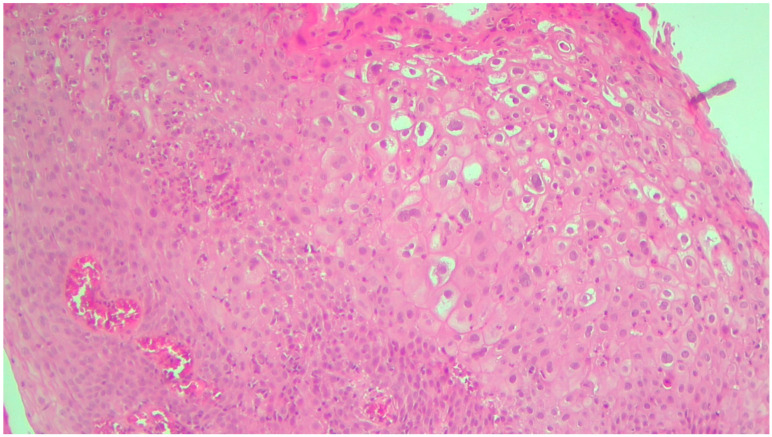
Large exophytic structures with papillary architecture lined by stratified squamous epithelium. HE ×100.

**Figure 6:**
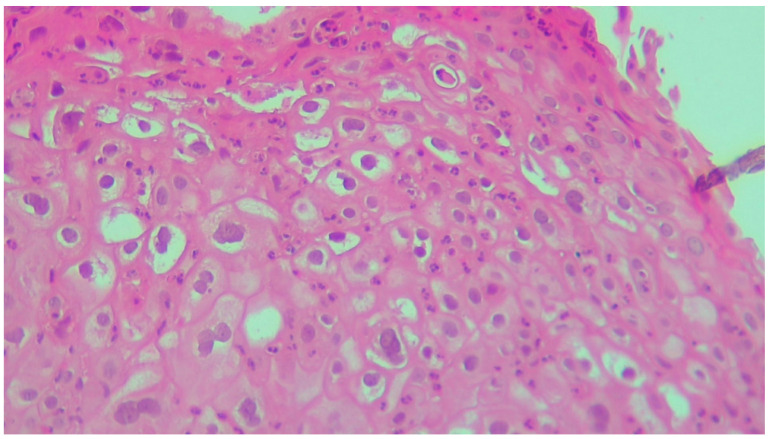
Squamous epithelium with koilocytes—cells with irregular nuclei, a perinuclear halo, and some with binucleation. HE ×200.

A 53-year-old heterosexual man, a smoker, with no hereditary-collateral background, presented in our department in June 2021 with a typical cauliflower-like tumor mass involving the perianal region, which had progressively increased in size for almost 7 years. (Figure 1 and Figure 2) The tumor resulted in issues related to bowel movements, constipation, and loss of control over passing gas and stool. These problems subsequently led to a lower food consumption and a gradual decline in body weight. The patient did not have any previous medical conditions or any surgery, nor medication use that would weaken the immune system. Local examination revealed a large vegetative lesion in the perianal area, a circular mass with a diameter of 13 × 9 cm, characterized by a verrucous texture, which encircled the anal area completely. There were no palpable locoregional lymph nodes. ECG and chest radiography showed no significant changes. Colonoscopy and anoscopy excluded involvement of the anal canal or sphincter. Bowel preparation was made with Fortrans (polyethylene glycols) the day before the surgery. The laboratory tests were normal and the patient was negative for human immunodeficiency virus. The results of viral serology indicated that the patient tested negative for HPV-16 and HPV-18. The perianal mass was completely removed, ensuring negative surgical margins, using a supramuscular plane approach. The procedure was conducted with the patient situated in a lithotomy position, and spinal anesthesia was administered, following the acquisition of written informed consent. The large perianal skin defect which occurred was reconstructed with a fascio-cutaneous V-Y advancement flap. (Figure 3) There was no need for protective stoma. Intravenous antibiotics, specifically cephalosporin and metronidazole, were administered for a duration of 7 days. In the early postoperative period, fecal contamination and physical activity were minimized, but the patient did not follow indications. A diet low in fiber was prescribed for the first week after the surgery. The importance of maintaining a healthy diet and discontinuing habits such as smoking and alcohol consumption were underlined. In spite of these efforts, a partial dehiscence of the wound still occurred on the third postoperative day and was left for secondary healing. The patient was released from the hospital on the 15th day, prior to the complete healing of the wound. Following the surgery, the patient experienced a significant enhancement in his subjective quality of life. This improvement primarily stemmed from the complete and thorough removal of the tumor, which reinstated their capacity to engage in everyday activities like sitting comfortably and having regular bowel movements. Furthermore, the patient regained his ability to interact with others without hindrance, and the fact that a stoma was not required also contributed to their improved well-being. After 5 months from the operation, the patient came back for a buttock abscess, which was incised and drained, the healing process taking a few weeks. After another 5 months, the patient returned for difficult defecation. The patient did not show up for his postoperative control after the first signs of difficult defecation, but when each fecal emission required great effort, the intestinal transit for feces was no longer daily. The patient did not have an absence of transit for gas, and there was no nausea or vomiting, only a loss of appetite. A clinical examination was conducted and anal stenosis was diagnosed. Immediately after establishing the diagnosis of late postoperative anal stenosis, the patient was guided to a gastroenterology center, where several pneumatic balloon dilatations were performed. The result of endoscopic treatment was ineffective, so surgery was elected. Under spinal anesthesia, an anal dilatation and sphinecterotomy were carried out, with good postoperative results. After 2 years from the last surgery, the patient presented for follow up, with no subjective complaints, and no pathological changes in clinical examination. (Figure 4) The histopathological result was ulcerated squamous papilloma. (Figure 5 and Figure 6)

The particularity of the case was the presence of a bulky perianal mass (BLT), which was excised with negative surgical margins. The patient developed one complication during the postoperative period, a partial dehiscence of the wound, which was treated conservatively, and two complications in the late follow-up, a buttock abscess and anal stenosis, both of them requiring surgery with a good postoperative evolution.

## 4. Conclusions

The timely identification of a Buschke–Löwenstein tumor, which primarily hinges on the patient’s consent, along with a dependable pathological diagnosis, is crucial for administering the right treatment and forecasting the treatment results. The surgical treatment of Buschke–Löwenstein tumors requires a multidisciplinary team with special expertise. The reconstruction techniques are difficult and may cause problems such as wound infection, dehiscence, hematoma, or tardive complications, such as anal stenosis or buttock abscess, which may require surgical intervention. There are many non-surgical treatments for this disease, but the majority are not efficient. The gold-standard treatment approach is surgical resection with clear margins and reconstruction in a single operation if the anal canal is involved in less than 50% of the circumference. Additional treatment is unnecessary to prevent postoperative relapses in Buschke–Löwenstein tumors.

We regard as standard treatment for BLT an aggressive surgery, which includes reconstructive surgery to uphold the best possible surgical outcomes and avert any recurrence.

## Data Availability

Not applicable.
